# Design and Modeling of a New Biomimetic Soft Robotic Jellyfish Using IPMC-Based Electroactive Polymers

**DOI:** 10.3389/frobt.2019.00112

**Published:** 2019-11-01

**Authors:** Zakai J. Olsen, Kwang J. Kim

**Affiliations:** Active Materials and Smart Living (AMSL) Lab, Department of Mechanical Engineering, University of Nevada Las Vegas, Las Vegas, NV, United States

**Keywords:** modeling, soft-robotics, biomimetics, ionic polymer-metal composites, electroactive polymers

## Abstract

Smart materials and soft robotics have been seen to be particularly well-suited for developing biomimetic devices and are active fields of research. In this study, the design and modeling of a new biomimetic soft robot is described. Initial work was made in the modeling of a biomimetic robot based on the locomotion and kinematics of jellyfish. Modifications were made to the governing equations for jellyfish locomotion that accounted for geometric differences between biology and the robotic design. In particular, the capability of the model to account for the mass and geometry of the robot design has been added for better flexibility in the model setup. A simple geometrically defined model is developed and used to show the feasibility of a proposed biomimetic robot under a prescribed geometric deformation to the robot structure. A more robust mechanics model is then developed which uses linear beam theory is coupled to an equivalent circuit model to simulate actuation of the robot with ionic polymer-metal composite (IPMC) actuators. The mechanics model of the soft robot is compared to that of the geometric model as well as biological jellyfish swimming to highlight its improved efficiency. The design models are characterized against a biological jellyfish model in terms of propulsive efficiency. Using the mechanics model, the locomotive energetics as modeled in literature on biological jellyfish are explored. Locomotive efficiency and cost as a function of swimming cycles are examined for various swimming modes developed, followed by an analysis of the initial transient and steady-state swimming velocities. Applications for fluid pumping or thrust vectoring utilizing the same basic robot design are also proposed. The new design shows a clear advantage over its purely biological counterpart for a soft-robot, with the newly proposed biomimetic swimming mode offering enhanced swimming efficiency and steady-state velocities for a given size and volume exchange.

## Introduction

Electroactive polymers (EAPs) have emerged and grown into a vast and diverse field of research, with numerous potential applications in soft robotics and smart materials. EAPs are a class of polymeric materials that respond to an external electrical stimulus, this includes size and shape changes which may be used in actuation (O'Halloran et al., 2008). The wide range of EAP materials may be divided into two categories, electronic EAPs, including ferroelectric polymers, dielectric elastomers, electro-strictive graft elastomers, and liquid crystal elastomers, and ionic EAPs, which include ionic polymer gels, ionic polymer-metal composites, conducting polymers, and others (Bar-Cohen, 2002; O'Halloran et al., 2008). In the model developed within this paper we will focus on the ionic polymer-metal composite (IPMC), though other actuator types may be just as easily used.

An IPMC consists of an ionic polymer, typically Nafion® or Aquivion® (Shahinpoor and Kim, 2001; Trabia et al., 2017), that is composited between two electrodes, most commonly platinum or gold. The IPMC material the capability of both electromechanical transduction, where they can act as actuators (Bonomo et al., 2007; Kim, 2007; Trabia et al., 2017), as well as mechanoelectrical transduction, where they work as sensors (Bonomo et al., 2006; Chen et al., 2007; Porfiri, 2009; Akle and Habchi, 2013). This duality lies in the fundamentals of the electrochemical nature that governs both transduction modes, and is explored throughout literature (Schicker and Wallmersperger, 2013; Cha and Porfiri, 2014; Shen et al., 2015a; Shahinpoor, 2015). As actuators, IPMCs have are capable of exhibiting large mechanical deformations in response to a relatively low voltage (Shahinpoor and Kim, 2001; Bar-Cohen, 2002; Wallmersperger et al., 2007; Jo et al., 2013; Shahinpoor, 2015), making them attractive for compact, low power soft robotics. Furthermore, their ability to actuate in water (Kim et al., 2007; Yim et al., 2007; Brunetto et al., 2008; Abdelnour et al., 2009) has focused the soft robotics development heavily on aquatic animals. The biomimetic applications of IPMCs range from small scale biological structures such as cilia (Sareh et al., 2012) all the way up to full size robots (Shen et al., 2015b).

The jellyfish has been the focus of many researchers in the biology and engineering field, with varying interests in its swimming mechanism. Here, a model for the swimming behavior of jellyfish is used to develop a new biomimetic soft robot design that builds on the basic mechanisms used in biology for locomotion and address any observed limitations. Biological jellyfish swim using one of two methods, rowing or jetting (Michael et al., 2013; Gemmell et al., 2015). The jet propulsion mechanism for locomotion consists of two distinct phases, the contraction and relaxation phase. During contraction, the jellyfish expels water out of an enclosed volume to generate thrust. To refill the fluid volume, the contraction phase is followed by a slower relaxation phase in which the internal volume takes in water through the velar aperture. During this process, a negative acceleration is experienced that slows the animal down. This will be the primary area of focus for developing a modified swimming mechanism for potential soft robotics.

Two approaches are presented and compared. First, a model that is rooted in a geometric description of a material body is used to gain qualitative information about the feasibility of a proposed robot design. After that, a refined mechanics model is proposed and developed. As will be shown throughout the paper, both the design of the biomimetic jellyfish robot as well as the approach taken for the modeling framework differ from what is found in literature. The design of the robot diverges from the traditional biomimetic robots in that the goal is not to take inspiration from the biological world and attempt to recreate a robot that might move or look in a similar fashion. Instead, inspiration from nature is used as a reference point, from which a new concept for locomotion is developed that only loosely mimics the principles found in biology.

## Methods

### Biological Inspiration

#### Kinematics of Swimming Jellyfish

**Figure 1:**
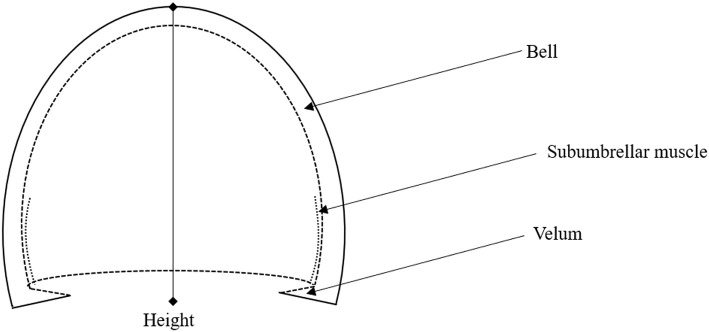
Illustration of jellyfish body structure, highlighting the bell, Subumbrellar muscle, velum, and height. The hemiellipsoid structure of the jellyfish body can be defined through the bell height and the velar aperture radius.

The jet propulsion mechanism used by jellyfish follows a simple equation of motion (EoM) that can be used to simulate the swimming behavior of these animals (Daniel, 1983). This equation also provides a starting point for modeling jellyfish-like robotics and their bioinspired swimming mechanisms. The swimming mechanism is broken down into four components: thrust, drag, inertia, and acceleration reaction. After a brief derivation found in Daniel (1983), the following EoM is obtained.

(1) $$\begin{array}{c} \left(1+\alpha_{AM}\right)\rho_{w}V_{f}\frac{du}{dt}=\frac{\rho_{w}}{A_{V}}{\left(\frac{dV_{f}}{dt}\right)}^{2}-{\frac{1}{2}C}_{d}\rho_{w}S_{A}u^{2}\; \end{array}$$

wherein α_*AM*_, ρ_*w*_, *V*_*f*_, *u*, *t*, *A*_*V*_, *C*_*d*_, and *S*_*A*_ are the added mass coefficient, fluid density, volume of fluid within the jellyfish body, linear velocity, time, velar aperture, drag coefficient, and cross-sectional area with respect to the direction of travel. The details of these parameters are given in Daniel (1983), and an illustration of the structure of a jellyfish is provided (Figure 1). Note that the direction of flow for the thrusting force is not reflected in this equation obtained from literature, but during implementation the thrust must be made positive during the contraction phase and negative during the relaxation phase due to the nature by which jellyfish perform their jet propulsion mechanism.

We take a brief moment to analyze this equation further. In the presented form, this equation is a result of applying Newton's second law, where we have taken the sum of the forces acting on the jellyfish, i.e., thrust, drag, and acceleration reaction, and related it to the acceleration, i.e., inertia, of the jellyfish. Here, only linear motion in one dimension is considered and the added mass is taken to be a scalar, greatly simplifying the problem. Nevertheless, we are still left with a non-linear equation due to the drag force being quadratic in the swimming velocity, in addition to the drag coefficient typically being a function of the flow conditions, and hence the swimming velocity. Furthermore, the volume and its rate of change, as well as the velar aperture and cross-sectional area, are dependent on the deformation of the jellyfish body and hence nearly all of the terms in Equation (1) are time dependent. Potential areas for simplification are cases in which the added mass is negligible or where its time variations may be ignored, as well as cases where the drag coefficient can be taken as constant or possibly as a small parameter suitable for perturbation techniques. In this paper, a few simplifying assumptions will be made regarding these parameters and will be discussed in detail as they arise.

**Figure 2:**
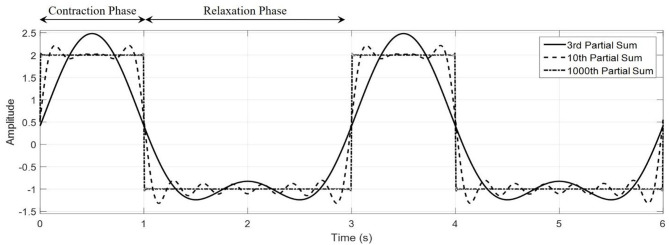
Representative waveforms generated with the Fourier series square wave. The characteristic contraction and relaxation phases of the jellyfish can be captured by the Fourier series shown here. Here, we have taken *t*_*c*_ = 1, *t*_*r*_ = 2, *A* = 2, *B* = −1.5, to create a purely illustrative waveform that demonstrate the role of these parameters.

As modeled in Daniel (1985), the volume rate of change is taken to be constant over both the contraction and relaxation phases. This can be achieved by defining a volume percentage change that should occur over these intervals, denoted Δ*V*, and calculating the rate of change over each phase as

(2) $$\begin{array}{c} \frac{dV_{f}}{dt}=\{\begin{array}{c} \begin{array}{cc} -\frac{\Delta V}{t_{c}} & contraction \end{array}\\ \begin{array}{cc} \frac{\Delta V}{t_{r}} & relaxation \end{array} \end{array}\; \end{array}$$

in which *t*_*c*_ and *t*_*r*_ are the durations of the contraction and relaxation phases, respectively. During contraction, the internal volume of fluid is ejected at rate given above and by performing this contraction over the duration *t*_*c*_, the volume changes by exactly −Δ*V*. Now by relaxing the bell, the internal volume of fluid may be filled at the relaxation rate, and by relaxing over a time *t*_*r*_ the bell volume increases by exactly Δ*V*, hence at the end of each swimming cycle the internal fluid volume returns to its initial value. This can be conveniently expressed using a variable amplitude square wave Fourier series of the form

(3) $$\begin{array}{l} F\left(t\right)\;=\frac{At_{c}+Bt_{r}}{t_{c}+t_{r}}\\ \;+\sum_{n=1}^{\infty }\frac{A-B}{n\text{π}}[\sin\left(\frac{2n\text{π}}{t_{c}+t_{r}}t_{c}\right)\cos\left(\frac{2n\text{π}}{t_{c}+t_{r}}t\right)\\ \;+\left(1-\cos\left(\frac{2n\text{π}}{t_{c}+t_{r}}t_{c}\right)\right)\sin\left(\frac{2n\text{π}}{t_{c}+t_{r}}t\right)] \end{array}$$

wherein *A* and *B* are the contraction and relaxation phase amplitudes, respectively. This type of input form will be used later when constructing two new models of soft-robotic systems, where it is of interest to see how the swimming behavior changes when this same input waveform is used for the volume rate of change, geometric parameters defining the bell, and electrical inputs to an IPMC model. Figure 2 illustrates how the waveform is structured, where during the contraction phase a larger volume rate of change occurs over a shorter period when compared to the relaxation phase. Using the Fourier series to describe the phenomena in Equation (2), the volume rate of change is expressed as

(4) $$\begin{array}{l} \frac{dV_{f}}{dt}=\overset{\infty }{\underset{n=1}{\sum }}\left(-\Delta V\right)\frac{t_{c}+t_{r}}{n\pi t_{c}t_{r}}[\sin\left(\frac{2n\pi }{t_{c}+t_{r}}t_{c}\right)\cos\left(\frac{2n\pi }{t_{c}+t_{r}}t\right)\;\\ \;+\;\left(1-\cos\left(\frac{2n\pi }{t_{c}+t_{r}}t_{c}\right)\right)\sin\left(\frac{2n\pi }{t_{c}+t_{r}}t)\right] \end{array}$$

This gives the volume of fluid contained within the jellyfish as

(5) $$\begin{array}{l} V_{f}=\;V_{0}-\overset{\infty }{\underset{n=1}{\sum }}\Delta V\frac{{\left(t_{c}+t_{r}\right)}^{2}}{2t_{c}t_{r}{\left(n\pi \right)}^{2}}[\cos\left(\frac{2n\pi }{t_{c}+t_{r}}\left(t-t_{c}\right)\right)\;\\ \;+\;1-\left(\cos\left(\frac{2n\pi }{t_{c}+t_{r}}t_{c}\right)+\cos\left(\frac{2n\pi }{t_{c}+t_{r}}t\right)\right)] \end{array}$$

for an initial volume of *V*_0_. Notice the term in Equation (3) outside of the summation does not appear in Equation (4), as this would give rise to a term that is linear in time in Equation (5). Elimination of this term from Equation (3) could be viewed as a kind of constraint on the parameters (*t*_*c*_, *t*_*r*_, *A, B*) to ensure that the integrated result oscillates about some initial value. With proper expressions for the cross-sectional area, drag coefficient, and added mass coefficient as provided in Daniel (1983), Equation (1) constitutes a first order non-homogeneous non-linear ODE in the swimming velocity variable *u*. The solution of this equation is easily obtained numerically via a state-space representation and 4th order Runge-Kutta integration to calculate position and velocity and integrate forward in time.

#### Design Principle of a New Biomimetic Jellyfish

As evident from the velocity profile found in Daniel (1983), the jellyfish swimming mechanism has a disadvantage due to the intake of water through the velar aperture during the relaxation phase. This causes a negative momentum exchange that pulls back on the animal, thus slowing it down. Here is where the proposed biomimetic robot seeks to modify the jet propulsion swimming mechanism. If the water that is drawn into the enclosed volume is redirected as to assist the propulsion of the device, then continual forward motion is achieved by an always increasing velocity until a steady state is reached. This, theoretically, should allow for a more efficient and effective swimming mechanism for a potential soft robot.

**Figure 3:**
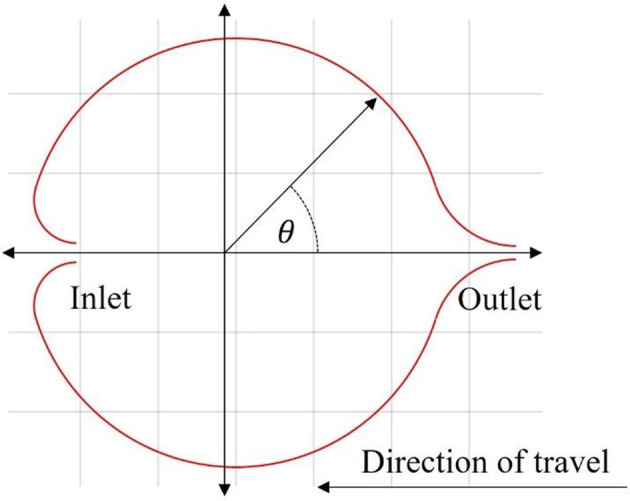
Illustration of the inlet and outlet valves. The direction cosine of an inlet or outlet for the proposed design is based off the polar angle starting on the positive x-axis and increasing in the counterclockwise direction.

To achieve this necessary modification, the addition of a distinct inlet and outlet to the enclosed volume is proposed. During contraction, only the outlet valve allows fluid flow, thus constraining the direction of the mass flux and hence the momentum exchanged. Then, during relaxation, the inlet would allow for the mass flux to occur along the same direction, therefore contributing a positive acceleration. A simple illustrative cross-section of such a design is given in Figure 3.

### Governing Equation and Model Inputs

In deriving Equation (1) there is an implicit assumption that the body of the jellyfish is approximately the same density as water. For arbitrary soft robot designs, this assumption is not valid and so the equation must be recast to account for variations in the density of the materials used to construct the robot as well as any potential onboard control devices. A simple modification is to split the robot's mass into a persistent mass that encompasses the materials that make up the structure and an internal volume of fluid contained within the body of the robot. Further, as added mass effects are by nature an interaction with the surrounding fluids, a distinction must be made between the interior and exterior volume of the robot. To account for the inlet and outlet directions as proposed in the design of this robot, a direction cosine is added to the thrust component. Hence, the equation of motion reads

(6) $$\begin{array}{lll} \left(m_{b}+\rho_{w}V_{i}+\alpha_{AM}\rho_{w}V_{o}\right)\frac{du}{dt} & = & \cos\left(\theta \left(t\right)\right)\frac{\rho_{w}}{A_{V}}{\left(\frac{dV_{i}}{dt}\right)}^{2}\\ \; & \; & -\frac{1}{2}C_{d}\rho_{w}S_{A}u^{2} \end{array}$$

with the mass of the robot body, *m*_*b*_, assumed to be fixed with a density not necessarily equal to water, the internal fluid volume, *V*_*i*_, the external volume of the body, *V*_*o*_, and the direction cosine, cos(θ(*t*)), illustrated in Figure 3, which is a function in time as the direction potentially changes for the contraction and relaxation phases. The direction cosine indicates the orientation of the flow through the inlet and outlet valves relative to the direction of forward travel. As mentioned briefly, in biological jellyfish the contraction phase expels fluid rearward through the velar aperture while the relaxation phase intakes fluid in the opposite direction, which in effect changes the direction of the thrust. Incorporating the direction cosine into the model allows for this alternating force to be directly reflected into the model. These modifications are necessary to capture a more accurate swimming behavior of the proposed robot. The time ratio of relaxation time to contraction time is defined in Equation (7).

(7) $$\begin{array}{l} \begin{array}{c} \delta_{t}=\frac{t_{r}}{t_{c}} \end{array} \end{array}$$

In the proposed model, the biomimetic robot has a forward-facing inlet that, under ideal circumstances, allows for unidirectional mass flow through the body of the robot, which will be referenced as the P1 swimming mode. This effect manifests itself in the direction cosine term in Equation (6). This kind of control in the model means it can also be used to simulate a jellyfish, P2, mode, in which the inlet is directed rearward, simulating the familiar swimming characteristics seen in Daniel (1983). The direction cosine angle for each mode is given in Table 1.

**Table 1:** Direction cosine angles for inlet and outlet during different swimming modes.

	**Propulsion mode 1 (P1)**	**Propulsion mode 2 (P2)**
Inlet	θ = 0	θ = π
Outlet	θ = 0	θ = 0

### Description of Robot Using a Geometric Model

**Figure 4:**
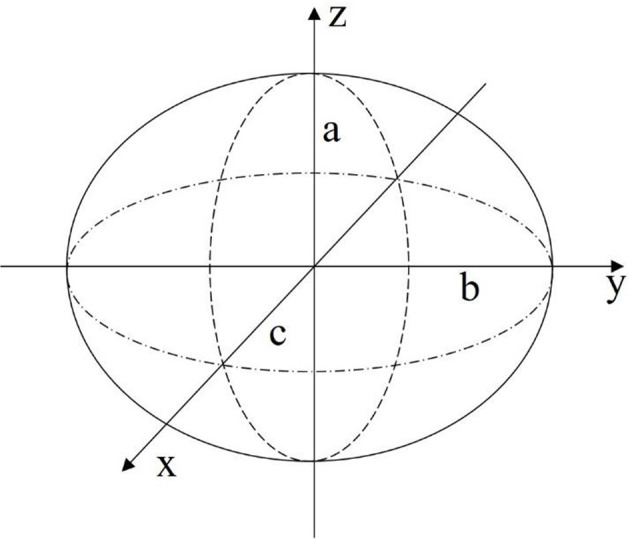
The body of the robot can be approximated as an ellipsoid for initial feasibility study of the proposed design.

The first modeling approach describes the body of the robot as a geometric surface. Specifically, the shell of the robot is defined as an ellipsoid with half-axis dimensions *a*, *b*, and *c* as illustrated in Figure 4. A constraint is placed on the model that all deformed states of the body can be described though the definition of an ellipsoid.

To use Equation (6) for the EoM, the volume, deformation of the volume, cross-sectional area, drag, and added mass effects must be determined. The internal volume of the given ellipsoid is calculated with Equation (8), and, with an added wall thickness of *d*, the external volume with Equation (9) below.

(8) $$\begin{array}{l} V_{i}=\frac{4}{3}\pi abc \end{array}$$

(9) $$\begin{array}{l} V_{o}=\frac{4}{3}\pi \left(a+d\right)\left(b+d\right)\left(c+d\right) \end{array}$$

The time rate of change for the internal and external volume are easily obtained by the chain rule for differentiation assuming that all three half-axis dimensions *a*, *b*, and *c* are all able to vary with time. For an input deformation of *a*, the corresponding deformations in *b* and *c* are obtained by enforcing conservation of mass and incompressibility with respect to the material contained between the internal and external volume. Assuming axisymmetric deformation about the z-axis, the rate of change in dimensions *b* and *c* in response to an input deformation to dimension *a* are found to be:

(10) $$\begin{array}{l} \frac{db}{dt}=\frac{dc}{dt}=-\frac{2b+d}{2\left(a+b+d\right)}\frac{da}{dt} \end{array}$$

The cross-sectional area with respect to the swimming direction can be calculated based on the external volume as

(11) $$\begin{array}{l} S_{A}=\frac{3V_{o}}{4\left(c+d\right)} \end{array}$$

The drag coefficient is calculated using the formulation found in Morrison (2013). This allows for a wide range of Reynold's numbers, 10^−1^ to 10^6^, and assumes a spherical body, for which this approach is valid under small deformations to an originally spherical geometry.

(12) $$\begin{array}{l} C_{d}\;=\;\frac{24}{Re}+\;\frac{2.6\frac{Re}{5.0}}{1+{\left(\frac{Re}{5.0}\right)}^{1.52}}+\;\frac{0.411{\left(\frac{Re}{263000}\right)}^{-7.94}}{1+{\left(\frac{Re}{263000}\right)}^{-8.00}}+\frac{0.25\frac{Re}{10^{6}}}{1+\frac{Re}{10^{6}}} \end{array}$$

Finally, the added mass coefficient for an ellipsoid body is found using (Korotkin, 2009).

(13) $$\begin{array}{l} A_{0}=abc\;\int_{0}^{\infty }\frac{du}{\left(c^{2}+u\right)\sqrt{\left(c^{2}+u\right)\left(b^{2}+u\right)\left(c^{2}+u\right)}}\;\\ \alpha_{AM}=\frac{A_{0}}{2-A_{0}} \end{array}$$

With these parameters fully defined, the EoM given in Equation (6) can be used to simulate the biomimetic robot swimming.

### Description of Robot Using a Mechanics Model

#### Equivalent Circuit Modeling of an IPMC

**Figure 5:**
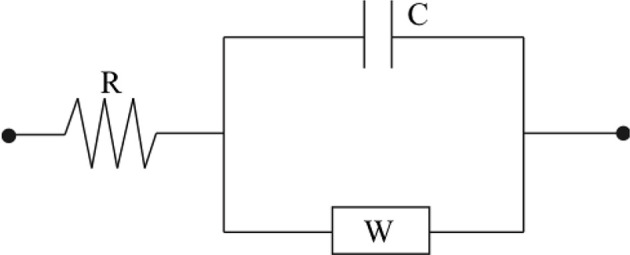
The circuit diagram can be used to construct an accurate model for the electromechanical transduction of IPMC actuators.

In developing a mechanics-based modeling approach a selection of actuator must be made. Here, the actuation model is that of an IPMC actuator, modeled through an equivalent circuit (EC). Similar to Shahinpoor and Kim (2001) and Cha et al. (2012), a circuit model that incorporates resistive, capacitive, and Warburg (RCW) impedances is used. These impedances model the surface and polymer resistance, inherent polymer capacitance and double layer capacitance due to cation migration, and charge transfer and diffusion within the polymer (Bard and Faulkner, 2000; Cha et al., 2012), respectively. A diagram of this circuit is provided in Figure 5.

The electrical impedance of each of these lumped circuit elements is written below, where *R*, *C*, and *W* are the lumped electrical resistance, lumped capacitance, and Warburg impedance, respectively

(14) $$\begin{array}{l} Z_{R}=R,\;Z_{Cap}=\;\frac{1}{j\omega C},\;Z_{Warburg}=\frac{W}{\sqrt{j\omega }} \end{array}$$

A transfer function may then be written between an input voltage and the respective current generated through the circuit (Cha et al., 2012).

(15) $$\begin{array}{l} H\left(s\right)=\frac{I\left(s\right)}{V\left(s\right)}=\frac{Cs+W\sqrt{s}}{RCs+RW\sqrt{s}+1} \end{array}$$

An electromechanical coupling equation can be written in the following form (Aureli et al., 2010)

(16) $$\begin{array}{l} P=\alpha Q \end{array}$$

where *P*, α, and *Q* are the mechanical loading of the IPMC, electromechanical coupling coefficient, and total charge within the polymer, respectively. Equation (16) links the electrochemical behavior of the IPMC from the EC model to the mechanical deformation of the IPMC which will be detailed later. The actuation response of an IPMC under the EC model can then be obtained from the charge via

(17) $$\begin{array}{lll} Q\left(t\right) & = & \int_{0}^{t}i\left(\tau \right)d\tau =L^{-1}\left\{\frac{1}{s}I\left(s\right)\right\}\\ \; & = & L^{-1}\{\frac{1}{s}H\left(s\right)L\left\{V\left(t\right)\right\}\} \end{array}$$

where *i*(*t*) is the electrical current through the circuit, V(t) is an external voltage applied to the IPMC electrodes, and L is the Laplace transform operator and an assumption of zero initial charge was made.

#### Deformation Modeling With Linear Beam Theory

**Figure 6:**
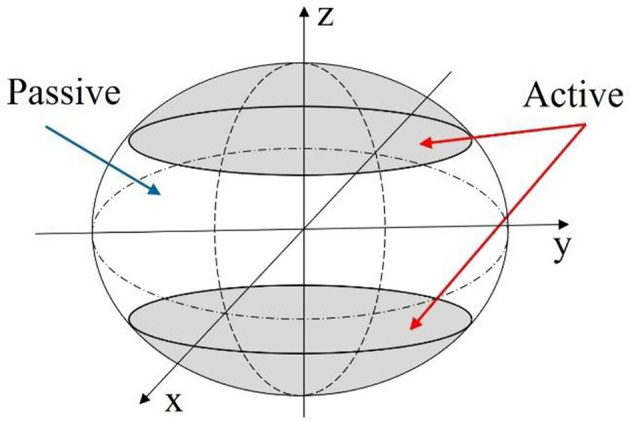
Diagram of the mechanics model description of the robot body. Active portions of the robot body can be deformed using a wide variety of EAP actuators.

**Figure 7:**
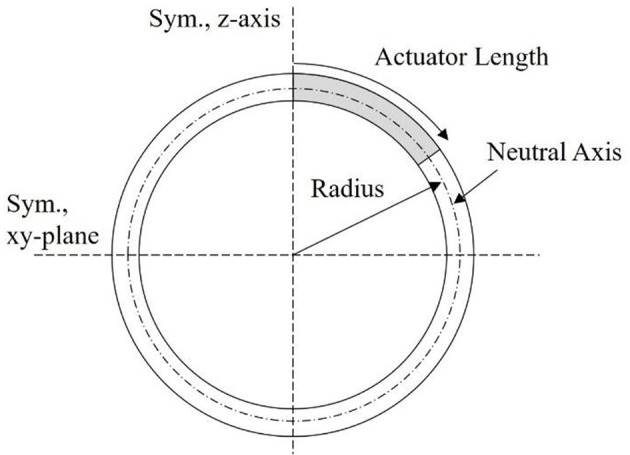
Illustrative cross-section of proposed mechanics model. The symmetry z-axis and xy-plane are highlighted, as well as the actuator length within the active portion of the shell wall, and the radius describing the neutral axis of the beam.

Relating back to Figure 4, the second modeling approach breaks away from the constraint of the robot body being defined by an ellipsoid geometry. Instead, as illustrated in Figure 6, the body is broken into active and passive regions, where the active region is physically deformed under the IPMC loading, and the passive region is dictated by the boundary conditions imposed on the geometry. Again, the deformation is assumed to be symmetric about the z-axis and across the xy-plane. This would be achieved by embedded IPMC actuators placed within the active region of the body and placed symmetrically around the z-axis. Other EAP actuators may be used in such a design, but here the model is restricted to just that of an IPMC. A cross-sectional view is provided in Figure 7 which highlights the placement of the active region of the shell as well as the plane and axis of symmetry.

To describe the deformation of the shell wall, a cross-section slice along the y-z plane is taken and the upper portion of the shell is modeled as a curved beam. Using virtual displacement, a functional of the form below is obtained

(18) $$\begin{array}{l} \int_{0}^{L}M\delta \kappa +N\delta \epsilon -P_{w}\delta w-P_{v}\delta vds=0 \end{array}$$

where *M*, *N*, *P*, *w v*, and *s* are the internal bending moment, internal axial load, external loading broken into transverse (*P*_*w*_) and axial (*P*_*v*_) directions, transverse and axial displacements, and the local axial coordinate along the length of the beam, respectively. The infinitesimal strain of the beam is defined in terms of the curvature and axial strain, κ and ϵ, given by

(19) $$\begin{array}{l} \kappa =w^{\prime \prime }+{(\frac{v}{\rho })}^{\prime } \end{array}$$

(20) $$\begin{array}{l} \epsilon =v^{\prime }-\frac{w}{\rho } \end{array}$$

wherein ρ is the undeformed radius of curvature of the beam, assumed to be constant for simplicity. The first terms in both strain expressions are the familiar axial and curvature strain found in the Euler-Bernoulli beam theory for straight beams (Byskov, 2013). The second terms are couplings of the transverse and axial deformation to the axial and curvature strains which is found only in curved beams. It can be easily verified that as the radius of curvature tends toward infinity, these terms tend to zero and thus the straight beam theory is recovered.

Now, the assumptions of an inextensible beam, transverse external load, and linear elasticity are taken which yields the new functional

(21) $$\begin{array}{l} \int_{0}^{L}EI\left(w^{\prime \prime }+\frac{w}{\rho^{2}}\right)\delta \left(w^{\prime \prime }+\frac{w}{\rho^{2}}\right)-P_{w}\delta wds=0 \end{array}$$

with Young's modulus *E* and cross-sectional moment of inertia *I* and the product *EI* is known as the flexural rigidity, or bending stiffness, of the beam. The Galerkin method is used to approximate the deflection in terms of monomials of the local axial coordinate, and is constructed as

(22) $$\begin{array}{l} \tilde{w}=\overset{N_{w}}{\underset{n=0}{\sum }}s^{n}{\hat{w}}_{n}=N_{s}\hat{w} \end{array}$$

where $\tilde{w}$ is the trial function, *N*_*w*_ is the number of monomial shape functions used, ***N***_**s**_ is a vector of the shape functions and $\hat{w}$ is a vector of the Galerkin coefficients ${\hat{w}}_{n}$. We may expand out the integrand of the functional and treat the variation on the displacement as a test function. Substitution of the Galerkin trial function into the functional in Equation (21) results in

(23) $$\begin{array}{l} \int_{0}^{L}[EI({N_{s}^{\prime \prime }}^{T}N_{s}^{\prime \prime }+\frac{1}{\rho^{2}}\left({N_{s}^{\prime \prime }}^{T}N_{s}+N_{s}^{T}N_{s}^{\prime \prime }\right)\\ \;+\frac{1}{\rho^{4}}N_{s}^{T}N_{s})\hat{w}-N_{s}^{T}P_{w}]ds=0 \end{array}$$

which is a symmetric system of linear equations to solve for the unknown Galerkin coefficients ${\hat{w}}_{n}$. The integration of the stiffness matrix is easily achieved using Gauss-Legendre quadrature. With the transverse deformation calculated from the Galerkin approximation, the condition of inextensibility can be used to determine the local axial deformation induced in the beam.

(24) $$\begin{array}{l} \epsilon =v^{\prime }-\frac{w}{\rho }=0 \end{array}$$

(25) $$\begin{array}{l} \tilde{v}=\frac{1}{\rho }\int_{0}^{s}\tilde{w}d\zeta =\frac{1}{\rho }\int_{0}^{s}\overset{N_{w}}{\underset{n=0}{\sum }}\zeta^{n}{\hat{w}}_{n}d\zeta =\frac{1}{\rho }\overset{N_{w}}{\underset{n=0}{\sum }}\frac{s^{n+1}}{n+1}{\hat{w}}_{n} \end{array}$$

The last step in this process illustrates how the use of monomial shape functions facilitates easier integration in the process of deriving these necessary equations. From here, the volume must be calculated based on the Galerkin approximation using

(26) $$\begin{array}{l} V=∭r^{2}sin\theta drd\theta d\varphi \end{array}$$

Equation (26) is integrated first with respect to the azimuthal angle, ϕ, and the radial distance, *r*, to obtain

(27) $$\begin{array}{l} V=\frac{4\pi }{3}\int_{\alpha }^{\beta }R^{3}sin\left(\theta_{s}\right)d\theta_{s} \end{array}$$

where the symmetry of the deformation has been leveraged and $\theta_{s}=\frac{s}{\rho }$ is the polar angle defining the position along the beam. The integration bounds are left as variable because the integrand, which is dependent on the Galerkin trial function, changes as the polar angle transitions from the active portion to the passive portion of the body. Substituting the trial function into the expression for radial position and factoring out the dependence on the loading and bending stiffness from the Galerkin coefficients yields

(28) $$\begin{array}{l} R=\left(\rho +\tilde{w}\right)=\left(\rho +\frac{P_{w}}{EI}\overset{N_{w}}{\underset{n=0}{\sum }}\theta_{s}^{n}{\tilde{\hat{w}}}_{n}\right) \end{array}$$

wherein a change of variable has been made from the local axial coordinate into the polar angle, and the Galerkin coefficients ${\hat{w}}_{n}$ have absorbed the dependence on ρ from this change of variable and become ${\tilde{\hat{w}}}_{n}$. The radial position is seen to have two components, the nominal radius of curvature with an addition of the transverse deflection along the length of the beam. Substitution of this into the volume integral results in

(29) $$\begin{array}{l} V\;=\frac{4\pi }{3}\int_{0}^{\frac{\pi }{2}}[\rho^{3}+3\rho^{2}\frac{P_{w}}{EI}\sum_{n=0}^{N_{w}}\theta_{s}^{n}{\tilde{\hat{w}}}_{n}+3\rho {\left(\frac{P_{w}}{EI}\sum_{n=0}^{N_{w}}\theta_{s}^{n}{\tilde{\hat{w}}}_{n}\right)}^{2}\;\\ \;+{\left(\frac{P_{w}}{EI}\sum_{n=0}^{N_{w}}\theta_{s}^{n}{\tilde{\hat{w}}}_{n}\right)}^{3}]\sin\left(\theta_{s}\right)d\theta_{s} \end{array}$$

The integration of the volume can be written in a compact form when noticing that the integrand, when expanded, is a series of monomials and sine products as coefficients of the loading and stiffness. The expansion and collection of these terms can be easily written in compact form, the results of which are given in the Supplementary Material. Finally, the internal volume is resolved into the cubic polynomial shown in Equation (30). The time dependence of the volume has now been highlighted and stems solely from the time variations in the loading, which in turn depends on the input voltage to the EC model of the IPMC as will be discussed shortly. The beam theory used has assumed static deflection, and thus the entire model is quasi-static, neglecting inertial effects in the mechanical deformation.

(30) $$\begin{array}{c} V\left(t\right)=A_{0}+A_{1}\left(\frac{P_{w}\left(t\right)}{EI}\right)+A_{2}{\left(\frac{P_{w}\left(t\right)}{EI}\right)}^{2}+A_{3}{\left(\frac{P_{w}\left(t\right)}{EI}\right)}^{3} \end{array}$$

The cross-sectional area with respect to the flow direction can be derived with a similar approach as that taken for the volume. Specifically, the area integral necessary is:

(31) $$\begin{array}{l} S_{A}=\iint r\;drd\theta_{s} \end{array}$$

With the radial distance defined in Equation (28), the integral becomes:

(32) $$\begin{array}{l} S_{A}=2\int_{0}^{\frac{\pi }{2}}\rho^{2}+2\rho \frac{P_{w}}{EI}\overset{N_{w}}{\underset{n=0}{\sum }}\theta_{s}^{n}{\tilde{\hat{w}}}_{n}\\ \;+{\left(\frac{P_{w}}{EI}\overset{N_{w}}{\underset{n=0}{\sum }}\theta_{s}^{n}{\tilde{\hat{w}}}_{n}\right)}^{2}d\theta_{s} \end{array}$$

The integral is in terms of only monomials of the polar angle thus its integration is relatively straight forward and can be evaluated to the expression below, with the coefficients *B*_0_, *B*_1_, and *B*_2_ provided in the Supplementary Material.

(33) $$\begin{array}{l} S_{A}\left(t\right)=B_{0}+B_{1}\left(\frac{P_{w}\left(t\right)}{EI}\right)+B_{2}{\left(\frac{P_{w}\left(t\right)}{EI}\right)}^{2} \end{array}$$

In the derivation of both volume and cross-sectional area the loading, *P*_*w*_, has been assumed constant along the length of the active portion of the beam. The added mass and drag coefficient, additional assumptions were made that allow the same relations of the geometric model to be used. The added mass was initially calculated based on an ellipsoid of largest volume that fit the material points along the x, y, and z-axis of the robot body. It was found that the added mass coefficient maintained very small oscillations around a value of 0.5, the value for a sphere, and due to the first approximation nature of this model the coefficient has been fixed to this value. Since the deformation to the body is relatively small, the assumption of a roughly spherical geometry is a reasonable approximation and hence drag is calculated again with Equation (12).

To couple the electromechanical transduction of an IPMC to the beam theory model, the mechanical loading of the beam, *P*_*w*_, is related to the electrochemical behavior of the IPMC through Equation (16). The Fourier series in Equation (3) is used as the model input here, where it defines the input voltage to the IPMC. An approximate inverse Laplace transform of Equation (17) is obtained using the FFT based NILT algorithm without acceleration, discussed in Brančik (2002) and Brancik and Smith (2015).

## Results

### Evaluation and Comparison of Models

**Figure 8:**
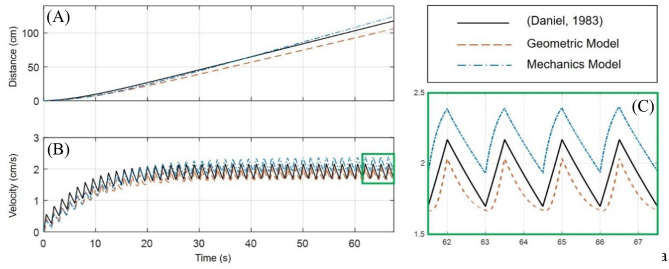
Comparison of the geometric and mechanics descriptions of the designed robot with a biological jellyfish model found in literature. The simulation uses a contraction time of 0.5 s, with a time ratio of 2. All models operate with ~10.5% volume exchange for thrust generation. **(A)** The position as calculated by the models for the jellyfish and robot show a slight curvature upward during the initial transient swimming phase and approach a nearly linear trend upon reaching steady-state swimming. **(B)** The initial curvature seen in **(A)** is seen to match the build up to steady-state swimming velocity in this plot. Initial transient swimming occurs approximately between 0 and 20 s, after which steady-state is reached and the velocity fluctuates around a steady value. **(C)** An enhanced portion of **(B)** is provided to more easily analyze the waveform of the velocity profiles. It is clearly seen that all three velocity profiles have similar sawtooth like characteristics, with differences in the curvature of the contraction and relaxation phases which is attributed to how the input signal induces deformation of the internal fluid volume.

A comparison of the geometric and mechanics models of the robot, in the P2 swimming mode, and an implementation of a biological jellyfish model from Daniel (1983) is provided in Figure 8. The biological jellyfish is modeled as being of comparable size and volume exchange to the biomimetic robots. For the jellyfish model a body length dimension of 23.1 mm was chosen with the remaining geometric factors scaling such that the enclosed volume matched that of the next two robot models. For the geometric model the three half-axis dimensions (*a, b, c*) defining the median surface of the ellipsoid body have an initial dimension of 31.75 mm, and a rate of change in the z-axis dimension of 40 mm/s during the contraction phase was chosen, with the relaxation phase being complementary such that the volume at the end of each swimming cycle returned to its initial state as discussed in section Kinematics of swimming jellyfish. The IPMC EC model used physical dimensions of 28 × 9.94 × 0.57 mm with a maximum input amplitude for the Fourier series of 3 V, with a body radius of 31.75 mm. The shell thickness was made to be 4 mm for both the geometric and mechanics model. All three models utilized a 10 mm velar aperture radius, volume exchange of 10.5%, contraction time of 0.5 s, and a time ratio of 2. Both the geometric and mechanics model assume a persistent mass of 50 g for the robot body, based on the amount of material that would be contained between the internal and external volumes and an approximate density, 1,065 kg/m^3^, of common castable silicon materials. The material properties of the IPMC were chosen from literature and use a Young's modulus value of 249 MPa (Trabia et al., 2017), while the shell was assumed to be made of castable silicon with an approximate modulus of 125 kPa. The composite structure for this thin bilayer cross-section was then transformed into an equivalent homogenous material cross-section using standard mechanics of materials approaches in order to calculate the bending stiffness used in the Galerkin approximation of the curved beam deformation.

The jellyfish model utilized Equation (1) for the governing EoM, with a volume rate of change given by Equation (2), or equivalently Equation (3). The remaining parameters are modeled just as in Daniel (1983). The geometric model uses the modified EoM of Equation (6), with the necessary volume, cross-section, drag, and added mass components obtained from Equations (8–13). For the mechanics model, the EoM of Equation (6) is again used, with a voltage input to the EC model, Equation (15), is obtained by a Fourier series of the form given in Equation (3). The charge response obtained from the EC model is used to calculate the mechanical loading through Equation (16), which in turn in controls the volume and cross-section from Equations (30) and (33), respectively.

An important note must be made here. The simulation of the biological jellyfish used was matched to a comparable volume exchange of the two proposed models. From Daniel (1983), the typical volume change for a jellyfish is on the order of 50%, where here a change of only 10.5% is used. While Figure 8 demonstrates that the two proposed biomimetic robot models do perform comparable to than the biological counter-part, the biological jellyfish model is not operating at full capacity. This limitation is due to the smaller overall deflection of the body in the mechanics models inherent in the small strain and small deformation assumption used. A more complex non-linear deformation model would allow for a wider range of volume changes to be simulated, but nevertheless the comparisons made here illustrate the swimming kinematics obtained under this new model.

From Figures 8A,B and it is clearly seen that all three of the models are operating with roughly the same performance, which is expected given they are exchanging the same amount of fluid and the inlet/outlet orientations are the same. One aspect to note is Figure 8C, which is an enhanced view of the boxed region of Figure 8B, in which the differences in the velocity profile during contraction and relaxation phases is seen. While the kinematics of the swimming for each model are nearly the same, being based on the two forms of the EoM, Equations (1) and (6), the differences in velocity profile are attributed to how the deformation to the internal fluid volume is obtained. In the standard jellyfish model based on the work in Daniel (1983), the internal volume of a truncated ellipsoid is changed at a constant rate during both the contraction and relaxation phases, as described by the Fourier series in Equation (4). For the geometric model, the z-axis dimension *a* is being used as an input, where the rate of change of *a* is driven by Equation (3), with the appropriately chosen values for the constants *A* and *B*. Lastly, the mechanics model uses the Fourier series of Equation (3), as the input voltage to the EC model of an IPMC, which then deforms the internal fluid volume according to the curved beam deformation calculated from the Galerkin approximation and Equations (30) and (33).

These three models use the same variable amplitude, variable duty cycle square wave to induce changes to the internal fluid volume through different means. It may be interesting to arrange each model to induce changes to the internal volume by the same means, such as constant rate of volume change, to directly compare the geometric differences between each of the approaches, but here we simply provide a brief discussion on the differences in the velocity profiles under the induced volume deformation described before.

**Figure 9:**
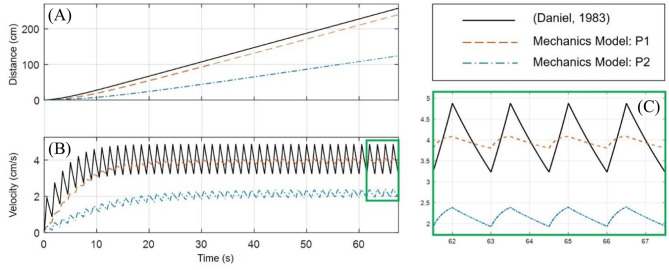
Comparison of the P1 and P2 swimming modes for the mechanics biomimetic robot model. The biological jellyfish as modeled according to Daniel (1983) must operate with nearly twice the volume exchange (20 vs. 10.5%) as the P1 biomimetic robot in order to obtain similar swimming performance. **(A)** As in Figure 8A, the position for the three simulations is plotted. The mechanics model in the P1 swimming mode far outpaces the P2 swimming mode, being on par with the biological model operating at a higher volume change. **(B)** The velocity profiles also show that the P1 mode has similar amplitude variations during steady-swimming as the P2 mode, but the biological model has larger variations for a roughly equivalent average steady-state velocity. **(C)** Again, an enhanced view of the velocity profiles at steady-state swimming is given which highlights the differences in the waveform generated by each model.

In Figure 9, the mechanics model is simulated under both the P1 and P2 swimming modes for the same 10.5% volume exchange. An implementation of the (Daniel, 1983) model is used to interpret the results, wherein a 20% volume exchange was necessary to obtain swimming performance similar to the biomimetic robot under the P1 swimming mode.

The Froude propulsion efficiency may be used to compare the jellyfish and biomimetic robot design ability to generate thrust during the contraction phase (Sfakiotakis et al., 1999; Ford and Costello, 2000; Moslemi and Krueger, 2010; Najem and Leo, 2012; Najem et al., 2012), and is calculated as

(34) $$\begin{array}{l} \eta_{P}=\frac{1}{t_{c}}\int \frac{2\;u\left(\tau \right)}{u\left(\tau \right)+v_{e}\left(\tau \right)}d\tau \end{array}$$

wherein *v*_*e*_ and *u* are the ejected fluid velocity, calculated by Equation (35) and robot velocity, respectively. The integral is taken over the duration of the final contraction phase after steady-state swimming is achieved. The efficiency results calculated for the simulations shown in Figures 8, 9 are given in Table 2.

(35) $$\begin{array}{l} v_{e}=\frac{\dot{m}}{\rho_{w}A_{v}} \end{array}$$

We find that at the simulated volume exchange of 10.5%, both the geometric and mechanics model of the biomimetic robot have propulsion efficiencies on the order of that of the simulated biological jellyfish. These values are reasonable when compared with jet propulsion studies found in literature (Bartol et al., 2009). Leveraging the forward-facing inlet in the P1 swimming mode, the biomimetic robot design can achieve a higher average velocity during steady-state swimming. This effectively makes each thrusting jet during contraction have a higher propulsive efficiency, as evident in the tabulated results. The higher propulsive efficiency achieved is on the same order as that obtained according to the biological jellyfish model of Daniel (1983) when operating with the much greater 50% volume change.

**Table 2:** Froude propulsion efficiency of each model is calculated for the 10.5% volume exchange simulations shown before.

**Volume exchanged**	**Jellyfish**	**Geometric**		**Mechanics**	
		**P1**	**P2**	**P1**	**P2**
10.5%	39.6%	69.9%	47.6%	62.5%	40.6%
50.0%	59.9%	N/A	N/A	N/A	N/A

*The biological jellyfish model reaches the ~60% propulsion efficiency range of the P1 swimming mode at a volume exchange of ~50%*.

### Energetics and Locomotive Cost for Biomimetic Robot

In Daniel (1983) the energetics of the jet propulsion for a model jellyfish are explored in order to characterize biological jellyfish and their locomotion. The locomotive cost is defined as

(36) $$\begin{array}{l} C=\frac{P_{i}}{Wu} \end{array}$$

wherein *P*_*i*_ is the rate of energy consumption, *W* the weight, and *u* the swimming velocity of the jellyfish. The locomotive efficiency is defined simply in terms of the output power over the input power.

(37) $$\begin{array}{l} \eta_{L}=\frac{P_{o}}{P_{i}} \end{array}$$

Since the jellyfish and the biomimetic robot design swim in unsteady patterns, the power input and output are averaged over appropriate swimming cycles. The power input is divided into the power required for generating a thrusting jet,

(38) $$\begin{array}{l} P_{i,t}=\frac{1}{t_{c}}\int Tv_{e}t \end{array}$$

which is averaged over the duration of a contraction phase, and the power required to expand and refill the internal volume

(39) $$\begin{array}{l} P_{i,f}=\frac{1}{t_{r}\left(1-\Delta \right)}\int Tv_{e}t \end{array}$$

which is averaged over the duration of a relaxation phase. The term (1 − Δ) represents the viscous loss through the deformation of the viscoelastic bell material in a jellyfish. Here, the dissipation factor Δ will be taken as zero, neglecting any losses through the deformation of the body material. The summation of these two terms gives the total average power input.

The power output is taken as the power required to overcome the effects of drag and inertia with added mass. These effects are averaged over the entire swimming cycle and are given below.

(40) $$\begin{array}{l} P_{o,d}=\frac{1}{t_{c}+t_{r}}\int \frac{1}{2}\rho_{w}S_{A}C_{d}u^{3}dt \end{array}$$

(41) $$\begin{array}{l} P_{o,a}=\frac{1}{t_{c}+t_{r}}\int \left(m_{b}+\rho_{w}V_{i}+\alpha_{AM}\rho_{w}V_{o}\right)\frac{du}{dt}u\;dt \end{array}$$

**Figure 10:**
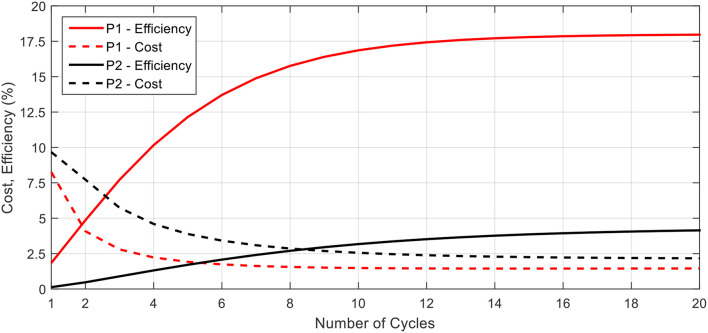
The locomotive cost and efficiency for the P1 and P2 swimming modes are presented here. In both cases, the efficiency increases while cost decreases as more swimming cycles are completed, and each parameter asymptotically approaches a steady state value. P1 curves for efficiency and cost are above and below, respectively, their corresponding P2 curves.

Using these equations, we may characterize the biomimetic robot design in terms of its locomotive efficiency and cost. Figure 10 provides the cost and efficiency plots for the proposed biomimetic robot as a function of the number of swimming cycles completed. The robot is swimming with a 0.5 s contraction time and a time ratio of 2. From the figure, a similar trend in cost and efficiency as found in the literature for biological jellyfish is seen. As the robot swims, the initial swimming cycles come at a higher cost and with a lower efficiency due to starting from rest and having to overcome the effects of inertia and added mass. As the robot approaches a steady state swimming, the cost and efficiency level off and the robot primarily fights against the drag forces as the average acceleration, and hence inertial effects, tend toward zero.

The data presented in Figure 10 also highlights the differences between the performance of the P1 and P2 swimming modes. For both the cost and the efficiency, the P1 swimming mode is seen to have better performance, starting and ending with lower cost and higher efficiency as compared to the P2 swimming mode. This, as well as the higher propulsive efficiency, may be attributed to the P1 modes capability to continually generate forward thrusting forces throughout the swimming cycle and hence capable of attaining higher mean velocities at steady state for the same amount of body deformation.

**Figure 11:**
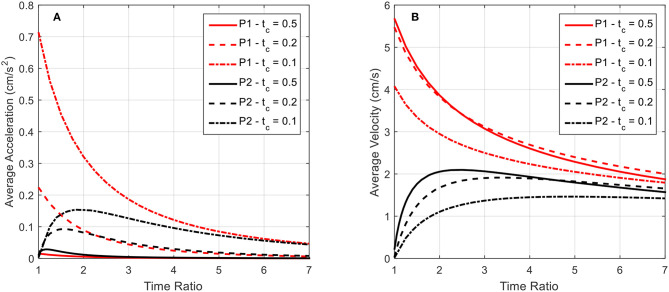
The average acceleration and average velocity after 20 swimming cycles is plotted as a function of the time ratio. Data for both P1 and P2 swimming modes is presented for contraction times of 0.5, 0.2, and 0.1 s. **(A)** As indicated by the above curves, increasing contraction time leads to a decrease in average acceleration after the 20 cycles for all time ratios, indicating that the robot has reached steady-state swimming, which is characterized by a zero average-acceleration. **(B)** In contrast to the average acceleration, the average velocity after the completed swimming cycles does not approach zero with increasing time ratio. As stated before, the near-zero average acceleration is indicative of approaching steady-state swimming, where a finite, non-zero velocity is expected.

In Figure 11, the average acceleration and average velocity over the final swimming cycle of twenty, as simulated in Figure 10, are plotted as a function of the time ratio for various contraction times. The results for the P2 swimming mode are similar in form to those found in Daniel (1983). A peak in average velocity is seen near the time ratio of 2, where the peak in acceleration shifts to lower time ratios as the contraction time is increased.

For the P2 swimming mode, the peak average acceleration is seen to decrease as the contraction time is increased, as opposed to the peak average velocity. To explain this, first consider that from the swimming dynamics we know during steady-state swimming, where the peak average velocity would be expected, the average acceleration per cycle should asymptotically approach zero. The lower peak acceleration seen in Figure 11A is then not necessarily indicating that the robot is generating lower peak accelerations during the contraction phase, but in fact is swimming in a state that is closer to its steady-state conditions, with a zero average acceleration. This implies that the robot has accelerated to this steady state faster within the 20 cycles that have been simulated, and hence is near its final average swimming velocity. This behavior is readily apparent if one were to examine the average acceleration as a function of the time ratio over a range of completed swimming cycles, wherein as the number of cycles increases the acceleration curves approach zero. The average velocity curves seem to be spread apart from each other based on the contraction time, but this may again be attributed to the fact that at the longer contraction times, the robot is accelerating to steady state in fewer swimming cycles, and hence is achieving a higher average velocity.

**Figure 12:**
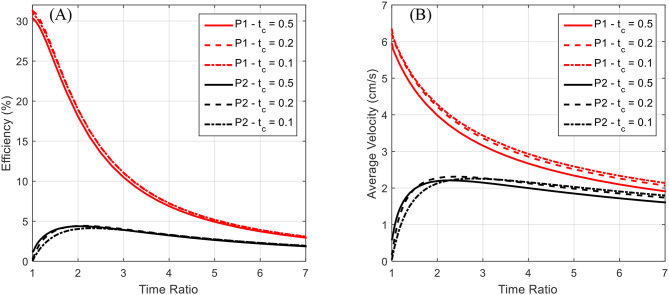
The locomotive efficiency and average velocity after reaching steady state is plotted as a function of the time ratio. Data for both P1 and P2 swimming modes is presented for contraction times of 0.5, 0.2, and 0.1 s. The P1 curves are above the corresponding P2 curves. **(A)** The locomotive efficiency during steady-state swimming is seen to decrease as a function of the time ratio, with the P1 swimming mode having a distinct advantage over P2. **(B)** A decrease in average steady-state swimming velocity with increasing time ratio is seen across all tested contraction times. Both P1 and P2 swimming modes appear to approach a general asymptote and decrease monotonically after a time ratio of ~3.

To glean more information, we turn to the steady state swimming results as presented in Figure 12. Here the locomotive efficiency and average velocity are presented as functions of the time ratio for various contraction times. Instead of using a consistent number of swimming cycles for each data point, these plots were generated by simulating the robot swimming until steady state was reached for each data point. Hence, average acceleration over a complete swimming cycle has approached zero and these curves are not presented.

Again, for the P2 swimming mode, which corresponds to normal jet propulsion found in the biological jellyfish considered, a peak in efficiency and average velocity is seen near a time ratio of 2 for all contraction times considered. This indicates that under normal jet locomotion, such a time ratio may be ideal in a wide range of circumstances. In both Figures 11B, 12B, we see that the average velocity for the P1 swimming mode is greater than that in the P2 mode. This is an expected result as the continual forward thrust for the same volume exchange should lead to a higher steady state velocity. Of greater interest is the behavior of these plots for smaller time ratios.

The plots seem to have a singular nature, increasing without bound as the time ratio approaches unity. Simulations were not carried out for time ratios of less than unity for two reasons. The first being that for a biological jellyfish, with time ratios less than unity the relaxation phase is performed over a shorter duration, and hence generates more negative thrust than positive thrust generated on the longer contraction phase. This in effect would result in backwards swimming, which is not of interest in this study. The second reason is that the current IPMC model does not allow for the frequency dependence on actuation amplitude to be accurately reflected. It is commonly found that as an IPMC, as well as other EAPs, are stimulated with higher frequencies, their actuation amplitude decreases. This would in effect generate a smaller volume exchange for the robot and deteriorate performance in the more rapid action regime of small contraction times and sub-unity time ratios. The lack of capability to capture these effects in the rapid actuation regime is due to the quasi-static nature of the beam deflection model. An introduction of inertial effects would allow for a more detailed analysis of the deformation dynamics but is not pursued further here. As indicated in Trabia et al. (2017), actuation decay for a typical IPMC actuating at 5 Hz, which would correspond to an actuation stroke of 0.2 s, is of the same order as one actuating at 1 Hz. Thus, the results for the short contraction time and small time ratio results given in Figures 11, 12 are reasonable as first estimations, but a more detailed dynamic analysis for the body deformation is needed in the future.

One final note of interest is that as seen in Figure 12 both the efficiency and the average velocity obtained at steady state nearly coincide for the various contraction times. This occurs in both the P1 and P2 swimming modes and may indicate that swimming performance is more so a function of overall geometric size, which governs the drag forces experienced in steady swimming, than the duration of the contraction phase.

### Device Design for Fluid Pumping and Thrust Vectoring

Aside from the use as a biomimetic jellyfish robot, the current design has other potential applications in the field of soft robotics. Visualizing the mass flux through the robot, during the contraction phase there is a negative flux as water leaves the internal volume. If a control volume is drawn at the outlet of the robot, and if the robot were constrained and fixed in place, the fluid motion through the control volume may be plotted. Assuming a forward-facing inlet as in the proposed design, the velocity through this control volume during the relaxation phase is zero. In this configuration the system now behaves as a unidirectional fluid pump.

**Figure 13:**
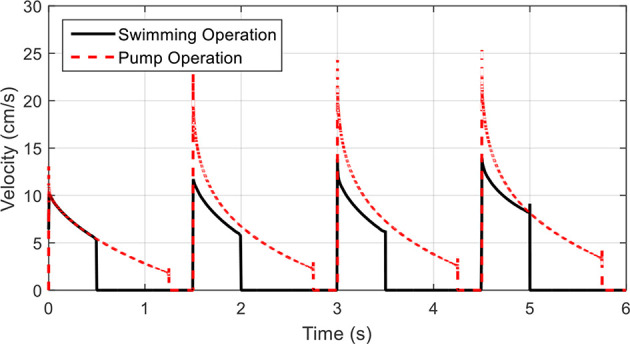
Velocity profiles for fluid pump operations. The outlet fluid velocity during a swimming operation shows the distinct pulse during contraction phases. By extending the contraction phase and shortening the relaxation phase, the outlet velocity becomes more continuous and the device may be used as a fluid pump.

For the kinematics described for swimming, where the time ratio between contraction and relaxation phases is larger than unity, the flow through this control volume pulsates. As the time ratio is lowered, the velocity through the control volume becomes more continuous. This is illustrated in Figure 13, where velocity profiles for the device in two regimes of contraction time/time ratio are shown. This application transforms the robot into a fluid pump, allowing for a near constant mass flow or a pulsating flow, making it suitable for multiple applications in low volume fluid pumping, akin to the proposed design in Lee et al. (2006). An interesting feature is the unidirectional aspect of this pump, where using one-way valves inherent in the structure of the shell, back flow is restricted. The IPMC driven actuation also makes the system a low voltage component. An alternative input voltage waveform may also allow for a more uniform flow velocity during the contraction phase but was not investigated in this study. The proposed design could further be used in thrusting applications where the device is not intended for self-locomotion as in the case of a biomimetic jellyfish, but for vectoring of a larger vehicle by providing a small thrusting force during a short contraction phase followed by a long relaxation phase that minimizes any additional forces.

## Discussion

### Key Findings

Designs and models for biomimetic jellyfish robots typically seek to mimic the structure of natural jellyfish, recreating the hemiellipsoid bell shape and deforming it with artificial muscles in place of the subumbrellar muscles. This approach has seen great success throughout literature (Yeom and Oh, 2009; Villanueva et al., 2010; Xiao et al., 2013), but the design presented here offers an alternative perspective on biomimetic soft robots. Without seeking to exactly mimic the functionality of the biological structure of the jellyfish, inspiration is taken from the physical mechanism of the locomotive jet propulsion used in some jellyfish. The freedom to design the inlet and outlet positions of a soft-robot allowed for the development of the alternative P1 swimming mode, which was shown to result in higher propulsive and locomotive efficiency as well as steady-state swimming velocity as compared to the biological model.

In comparing this design model with some other biomimetic jellyfish robots in literature, we find that the predicted results are in good agreement with the experimental performances. The design found in Xiao et al. (2013) utilizes more traditional robotic actuators to locomote with the P2 swimming of biological jellyfish, and their experiments gave a range of 3–8 cm/s for the forward swimming speed and 1 cm/s during a diving and surfacing operation which are very compatible to the results obtained here. The authors of Nawroth et al. (2012) showed their biomimetic medusoid swimming with a velocity of approximately 0.5 body lengths per stroke (BL/S), while a biological jellyfish had an average near 0.78 BL/s. When the results provided in Figure 8 are converted into this framework we find the geometric and mechanics models operating at a velocity of 0.14 and 0.18 BL/S, respectively, while the results of Figure 9 for the jellyfish model and the P1 mechanics model give 0.86 and 0.31 BL/S, respectively, which fit nicely with the results of Nawroth et al. (2012). The authors in Villanueva et al. (2010) and Villanueva et al. (2011) created a biomimetic jellyfish using shape memory alloy materials and demonstrated swimming velocities, with similar characteristics and shown here, in the range of 1–3 cm/s during initial startup swimming, in line with the results of the mechanics model in the P1 swimming mode. Using IPMC actuators, Najem et al. (2012) created a biomimetic jellyfish robot that utilizes a locomotive method more akin to the paddling mode of biological jellyfish, which is expected to generate less deformation of the enclosed fluid and hence less thrust, and achieved experimental speeds in the range 0.36–1.5 mm/s, which are lower than predicted here. This may be attributed to the fact that in the models presented here a small velar aperture is used, which generates a higher thrusting force according to Equation (6), and hence achieves larger average velocities. Overall, the model presented here predicts results that are in line with existing literature and demonstrate the utility of the new design and its capability to utilize a more effective and efficiency swimming method (P1).

As already discussed, the current mechanics model relies on a quasi-static linear beam deformation, which is inadequate for capturing the dynamic nature of the body deformation. Because of this, further investigation on the high frequency actuation for both the P1 and P2 swimming modes is needed. Furthermore, the simple square wave input used for each model may not be ideal for optimizing locomotive efficiency, steady-state velocity, or even initial transient acceleration. The effects of input waveform for each model on the deformation of the internal fluid volume is another avenue of research. Despite this, the newly proposed design shows promise for a novel biomimetic jellyfish robot which utilizes a unique propulsive mechanism for locomotion. The new design also shows promise as a low power, low volume fluid pumping mechanism that utilizes soft EAP actuators.

While this new approach to locomotion is theorized to provide a higher degree of swimming efficiency, it is clearly not the method jellyfish have adapted into their structure. One such reason may be due to the more complex muscular structure required to maintain two apertures that must operate in a synchronous fashion to provide any thrust at all. Any additional efficiency might simply not outweigh the cost of the added complexity. Furthermore, as discussed in Daniel (1983), jellyfish might not have evolved to simply maximize locomotive efficiency or steady-state velocity. Other factors such as their capability to avoid prey or better source food from the environment might have a larger role that makes the design proposed here ill-suited for the biological world. The field of soft robotics however is free of such biological imperatives, and as such the proposed design and P1 swimming mode offer a new perspective on the biomimetic approach for robot development.

## Conclusion

Biology has been shown to provide invaluable inspiration for the modeling, design, and development of soft robotic systems. Here, insight of the jet propulsion mechanism found in jellyfish lead to a new theory about an improved swimming mechanism for small aquatic robots. This work demonstrated the effects of redirecting the fluid intake direction in a jellyfish type swimming mechanism through two different modeling approaches. The geometric description the proposed robot design is an idealized simplification that gives insight into some of the swimming behaviors of such design and serves as a reasonable approach for conducting initial feasibility studies on new conceptual robot designs. To bring the model closer to reality, the physic-based model used linear beam theory and equivalent circuit models of EAP actuators to construct a new approach to soft-robotic modeling that can be easily expanded to increase accuracy. Both models demonstrated the potential improvements of the P1 swimming mode as compared to that of a typical jellyfish. This new design shows promising applications for biomimetic soft robotics as both an aquatic swimming robot as well as a device for fluid pumping or thrust vectoring.

## Data Availability Statement

The datasets generated for this study are available on request to the corresponding author.

## Author Contributions

ZO performed the study under supervision of KK. ZO wrote the manuscript. KK reviewed the manuscript.

### Conflict of Interest

The authors declare that the research was conducted in the absence of any commercial or financial relationships that could be construed as a potential conflict of interest.
