# Supplementary material for: Design and Modeling of a New Biomimetic Soft Robotic Jellyfish Using IPMC-Based Electroactive Polymers
Source: Front Robot AI. 2019 Nov 1;6:112. doi: 10.3389/frobt.2019.00112 (PMC7805721; doi:10.3389/frobt.2019.00112)
Supplement: Supplementary file 1 [file Data_Sheet_1.DOCX]

Supplementary Material

# Supplementary derivation of volume and cross-sectional area coefficients

The integrand of Equation (29) can be expanded into a set of integrals of the form below.

$$\begin{aligned} \mathcal{I}_{n}\left( \theta\right)=\int_{\alpha}^{\beta} \theta^{n}\sin\theta d\theta\#\left( S.1 \right) \end{aligned}$$

Using integration by parts, the integral can be shown to be equal to the following series representation for the indefinite integral,

$$\begin{aligned} \mathcal{I}_{n}\left( \theta\right)=\cos\left( \theta\right)\sum_{m=0}^{\left\lfloor\frac{n}{2} \right\rfloor} \frac{\left( -1 \right)^{m+1}n!}{\left( n-2m \right)!}\theta^{n-2m}+sin \left( \theta\right)\sum_{k=0}^{\left\lfloor\frac{n-1}{2} \right\rfloor} \frac{\left( -1 \right)^{k}n!}{\left( n-2k-1 \right)!}\theta^{n-2k-1}\#\left( S.2 \right) \end{aligned}$$

where $\left\lfloor\right\rfloor$ is the floor operator. This allows for definite integrals of the form (S.1) to be written in a compact notation

$$\begin{aligned} I_{n}\left( \alpha;\beta\right)=\mathcal{I}_{n}\left( \beta\right)-\mathcal{I}_{n}\left( \alpha\right)\#\left( S.3 \right) \end{aligned}$$

The integration of the internal volume can now be written in a polynomial form in terms of this expression. The expansion and collection of these terms can be easily written using summations, the results of which are given below for the integral coefficients found in the manuscript.

$$\begin{aligned} A_{0}=\frac{4\pi}{3}\rho^{3}\#\left( S.4 \right) \end{aligned}$$

$$\begin{aligned} A_{1}=4\pi\rho^{2}\sum_{n=0}^{N_{w}} I_{n}\left( 0;\frac{\pi}{2} \right){\tilde{\hat{w}}}_{n}\#\left( S.5 \right) \end{aligned}$$

$$\begin{aligned} A_{2}=4\pi\rho\sum_{m=0}^{N_{w}} \sum_{n=0}^{N_{w}} I_{n+m}\left( 0;\frac{\pi}{2} \right){\tilde{\hat{w}}}_{m}{\tilde{\hat{w}}}_{n}\#\left( S.6 \right) \end{aligned}$$

$$\begin{aligned} A_{3}=\frac{4\pi}{3}\sum_{l=0}^{N_{w}} \sum_{m=0}^{N_{w}} \sum_{n=0}^{N_{w}} I_{n+m+l}\left( 0;\frac{\pi}{2} \right){\tilde{\hat{w}}}_{l}{\tilde{\hat{w}}}_{m}{\tilde{\hat{w}}}_{n}\#\left( S.7 \right) \end{aligned}$$

The integration of Equation (32) more straight forward since there is no transcendental term. Thus, the integral coefficients of Equation (33) are easily obtained as

$$\begin{aligned} B_{0}=\pi\rho^{2}\#\left( S.8 \right) \end{aligned}$$

$$\begin{aligned} B_{1}=4\rho\sum_{n=0}^{N_{w}} \frac{\left( \frac{\pi}{2} \right)^{n+1}}{n+1}{\tilde{\hat{w}}}_{n}\#\left( S.9 \right) \end{aligned}$$

$$\begin{aligned} B_{2}=2\sum_{m=0}^{N_{w}} \sum_{n=0}^{N_{w}} \frac{\left( \frac{\pi}{2} \right)^{n+m+1}}{n+m+1}{\tilde{\hat{w}}}_{n}{\tilde{\hat{w}}}_{m}\#\left( S.10 \right) \end{aligned}$$
